# Dynamic screening of a localized hole during photoemission from a metal cluster

**DOI:** 10.1186/1556-276X-7-447

**Published:** 2012-08-08

**Authors:** Natalia E Koval, Daniel Sánchez-Portal, Andrey G Borisov, Ricardo Díez Muiño

**Affiliations:** 1 Centro de Física de Materiales CFM/MPC (CSIC-UPV/EHU), Paseo Manuel de Lardizabal 5, San Sebastián, 20018, Spain; 2 Donostia International Physics Center DIPC, Paseo Manuel de Lardizabal 4, San Sebastián, 20018, Spain; 3 Institut des Sciences Moléculaires d’Orsay, ISMO, Unité de Recherches CNRS-Université Paris-Sud UMR 8214, Bâtiment 351, Université Paris-Sud, Orsay Cedex, 91405, France

**Keywords:** Spherical jellium cluster, Time-dependent density functional theory, Dynamic screening, Photoemission

## Abstract

Recent advances in attosecond spectroscopy techniques have fueled the interest in the theoretical description of electronic processes taking place in the subfemtosecond time scale. Here we study the coupled dynamic screening of a localized hole and a photoelectron emitted from a metal cluster using a semi-classical model. Electron density dynamics in the cluster is calculated with time-dependent density functional theory, and the motion of the photoemitted electron is described classically. We show that the dynamic screening of the hole by the cluster electrons affects the motion of the photoemitted electron. At the very beginning of its trajectory, the photoemitted electron interacts with the cluster electrons that pile up to screen the hole. Within our model, this gives rise to a significant reduction of the energy lost by the photoelectron. Thus, this is a velocity-dependent effect that should be accounted for when calculating the average losses suffered by photoemitted electrons in metals.

## Background

Photoemission spectroscopy is one of the most important techniques used to study the structure of molecules, surfaces, and solids [1]. It is based on the photoelectric effect which was discovered more than 100 years ago by H. Hertz. Later, in 1905, Albert Einstein explained this effect as a quantum phenomenon, based on the emission of electrons from a target following the absorption of a photon (‘quantum of light’). Photoemission spectroscopy has significantly contributed to the understanding of fundamental principles in solid state physics.

In the recent years, progress in laser technology has made possible the development of photoemission spectroscopy in the attosecond range (1 as = 10^−18^s) [2]. Attosecond techniques permit access to the time scale of electron motion in atoms, molecules, and solids. Due to this experimental advance, there is a growing interest in the theoretical description of the dynamic electronic processes taking place in the subfemtosecond time scale [3-6].

In the present work, we study the electron dynamics during photoemission from small metallic clusters (or nanoparticles). Clusters represent a bridge between individual atoms and solid state materials [7]. Due to their large surface to bulk ratio, small metal clusters can exhibit rather unique features. For example, they frequently present interesting catalytic properties. Our choice of a finite-size system as target in the photoemission process simplifies the theoretical analysis, but some of our conclusions are expected to remain valid in extended systems such as metal surfaces.

We consider the case where one of the atoms in the metallic cluster undergoes core-electron photoemission. We focus our attention on the combined dynamic screening of a static localized core hole and the photoemitted electron. We show that the presence of the hole left behind affects the many-body electronic dynamics in the cluster and therefore the emission dynamics of the photoelectron. For the description of the many-body response of the valence electrons in the cluster, we use time-dependent density functional theory (TDDFT) - an *ab initio* quantum-mechanical method. Our TDDFT methodology has already been successfully applied to study the dynamic screening of charges in finite-size systems [8,9] and to the calculation of the energy transfer between particles and small gas-phase clusters [10,11]. In the present calculations, the motion of the photoemitted electron is described classically. This approximation is justified provided that typical energies of the photons are in the 100 eV range of extreme ultraviolet (XUV), resulting in relatively high energies of the photoemitted electrons, as the ones considered here. To analyze the role of the many-body screening effects, we perform calculations using various approximations for the classical trajectory of the electron, including constant velocity studies and calculations with and without direct interaction between the ejected electron and the hole left behind.

The problem we are addressing here has a long history in condensed matter physics. The dynamic relaxation of the Fermi sea after a creation of a hole was analyzed in the context of X-ray photoemission by several authors [12,13]. Within the framework of linear response theory, Noguera et al. showed that the effective interaction between the core hole and the photoemitted electron changes continuously from a statically screened potential for low-energy electrons to a completely unscreened potential for high-energy electrons [13]. They also showed that the double screening of hole and electron can occur with or without creation of plasmons according to the kinetic energy of the emitted electron. Here we go beyond linear theory in the description of the dynamic screening of charges in the photoemission process by using propagation of electronic wave packets with TDDFT to compute the response of the valence electrons.

## Methods

In the present study, metallic clusters are described using a spherical jellium model (JM). Despite its simplicity, the JM can be very useful in the interpretation of photoemission data from metal clusters, as recently shown in [14]. In the JM, the core ions are substituted by a homogeneous background of positive charge with a density defined by

(1) $$n_{0}^{+}(\text{r})=n_{0}(r_{s})\Theta (R_{\text{cl}}-r),$$

where *R*_cl_ is the radius of the cluster, *Θ*(*x*) is the Heaviside step function and *n*_0_(*r*_s_) is the constant bulk density, which depends only on the Wigner-Seitz radius *r*_s_( $1/n_{0}=4\Pi r_{s}^{3}/3$) [15]. The latter is the only parameter in the JM. The number of electrons in a neutral cluster is $N={\left(R_{\text{cl}}/r_{s}\right)}^{3}$. For simplicity, we only consider closed-shell clusters in our calculations.

In order to obtain the ground state electronic density of the cluster *n*(**r**), we use the spin-restricted density functional theory [16] and solve the Kohn-Sham (KS) equations [17]:

(2) $$\left\{-\frac{1}{2}\nabla^{2}+V_{\text{eff}}([n],\text{r})\right\}\varphi_{i}=\varepsilon_{i}\varphi_{i},$$

where *ε*_i_ are the eigenvalues of the KS equations and *φ*_i_ are the one-electron wave functions. Please notice that unless otherwise specified, we use Hartree atomic units (a.u.) throughout the paper. The effective potential is composed of three terms:

(3) $$V_{\text{eff}}([n],\text{r})=V_{\text{ext}}(\text{r})+V_{H}(\text{r})+V_{\text{xc}}(\text{r}),$$

where *V*_ext_(**r**) is the external potential created by the positive background, *V*_H_(**r**) is the Hartree (or Coulomb) potential created by the electronic density, and *V*_xc_(**r**) is the exchange-correlation potential, calculated in our case in the local-density approximation with the Perdew-Zunger parametrization of Ceperley-Alder exchange and correlation potential [18].

**Figure 1:**
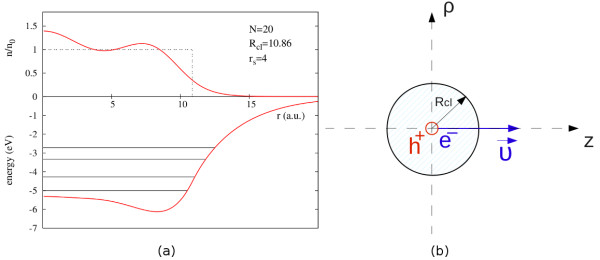
**Ground state electronic density, effective Kohn-Sham potential, and sketch of the photoemission process.** (**a**) Upper panel, ground state electronic density in units of the positive background density (dashed line, *r*_*s *_= 4) for a cluster containing 20 electrons; lower panel, the corresponding effective Kohn-Sham potential and occupied energy levels. (**b**) Sketch of the photoemission process. An electron and a hole are created at the center of the spherical cluster at *t *= 0. Both are represented by classical point particle and the electron starts to move along the *z*-axis with velocity *υ*_0_.

The electronic density *n*(**r**) is given by a sum over occupied wave functions:

(4) $$n(\text{r})=2\underset{i\in \text{occ}}{\sum }|\varphi_{i}(\text{r}){|}^{2},$$

where the factor 2 stands for spin degeneracy. Results for the ground state of small metal clusters have been thoroughly discussed in the literature [19-21]. The effective KS potential and the ground state electronic density for one of the clusters considered here are shown in Figure 1a.

For the description of the photoemission process, we use a semi-classical model. We consider fast photoemitted electrons that are moving with velocities much higher than the Fermi velocity of the cluster electrons. Therefore, the movement of the photoelectron can be represented classically. In parallel, valence electron dynamics in the cluster is investigated by means of TDDFT [22]. In TDDFT, the evolution of the electronic density *n*(**r**), in response to the field of the moving electron, is calculated by solving the time-dependent KS equations:

(5) $$\;\begin{array}{l} i\frac{\partial }{\mathrm{\partial t}}\varphi_{i}(r,t)=\left\{-\frac{1}{2}\nabla^{2}+V_{\text{ext}}(\text{r})+V_{H}(\text{r},t)+V_{\text{xc}}(\text{r},t)\right)\\ \;\;+△V(\text{r},t)\}\varphi_{i}(\text{r},t), \end{array}$$

where Δ*V*(**r***t*) is the change of the external potential due to the photoemission process (see discussion below). The exchange-correlation potential *V*_xc_ is calculated with the standard adiabatic local density approximation with the parametrization in [18].

The time-evolving electronic density of the excited cluster is obtained from the time-dependent KS orbitals *φ*_i_(**r**,*t*), in a way similar to Equation 4. The time-dependent KS wave functions are obtained by propagating the initial wave functions *φ*_i_(**r**,*t*_0_) =* φ*_i_(**r**) using the split-operator technique. Due to the presence of the photoemitted electron, the problem loses its spherical symmetry and the use of cylindrical coordinates (*ρz*) becomes necessary. A detailed description of the numerical procedure can be found in [23-25].

In our model, we do not consider explicitly the interaction with an external electromagnetic field. Thus, an electron with high kinetic energy and a static hole are created at *t *= 0 at the center of the spherical cluster. The scheme of the process is shown in Figure 1b. The photoemitted electron (el) is modeled as a negative point charge that moves along the *z*-axis [*ρ *= 0, *z*_el_(*t*)]. It is worth noting that this photoemitted electron is not one of the cluster valence electrons, but an extra electron coming from the inner shell of a hypothetical atom sited in the center of the cluster. The electron motion is calculated in two different approximations as described in detail below. The hole (*h*) is represented by a positive point charge at a fixed position [*ρ *= 0, *z*_*h *_= 0]. The potential created by these charges and acting on the rest of the electrons in the cluster is Δ*V *=* V*_el_ + *V*_*h*_, where

(6) $$V_{\text{el}}=\frac{1}{{\left[{\left(z_{\text{el}}(t)-z\right)}^{2}+\rho^{2}\right]}^{1/2}}\Theta (t),$$

and

(7) $$V_{h}=-\frac{1}{{\left[z^{2}+\rho^{2}\right]}^{1/2}}\Theta (t).$$

To address the effect of the many-body dynamics in the cluster on the energy loss experienced by the ejected electron in well-defined conditions, we first study a simplified case in which the photoemitted electron is assumed to move with constant velocity *υ*, i.e., *z*_el_(*t*) =* υt*. This allows us to isolate the effects related to the dynamics of the screening processes from other possible effects associated with the details of the trajectory. Here *υ*corresponds to the final velocity of the electron if the photoemission process would take place in vacuum, which is considered as a good approximation for the average electron velocity during its movement through the cluster. Thus, the direct interaction between the electron and the hole is not taken into account in this case. However, as we will see below, the screening of the hole still has an important influence on the energy loss by the photoemitted electron. In this case the energy loss is calculated from the integral

(8) $$E_{\text{loss}}=-\upsilon \overset{\infty }{\underset{0}{\int }}F_{z}^{\text{cls}}(t)\;\text{dt},$$

where

(9) $$\;F_{z}^{\text{cls}}(t)=2\Pi \int \text{dρdz}\;\rho \frac{n(\rho ,z,t)-\overset{+}{\underset{0}{n}}(\rho ,z)}{{\left[{\left(z_{\text{el}}(t)-z\right)}^{2}+\rho^{2}\right]}^{3/2}}[z_{\text{el}}(t)-z]$$

is the *z* component of the force created by the cluster on the emitted electron. It is important to note that *E*_loss_includes the energy necessary to eject the electron from the cluster (an adiabatic contribution), as well as nonadiabatic contributions due to the creation of electronic excitations in the cluster during the emission process.

In the second step, we perform a more refined treatment in which the direct electron-hole interaction is included and the trajectory *z*_el_(*t*) is calculated using the classical equations of motion:

(10) $$\begin{array}{l} dz_{\text{el}}/\text{dt}=\upsilon (t),\;z_{\text{el}}(t=0)=0\; \end{array}$$

(11) $$\begin{array}{l} \mathrm{d\upsilon}/\text{dt}=F_{z}^{\text{tot}}(t),\;\upsilon (t=0)=\upsilon_{0}.\; \end{array}$$

In Equations 10 and 11, $F_{z}^{\text{tot}}$ is the total force felt by the moving electron

(12) $$F_{z}^{\text{tot}}(t)=F_{z}^{\text{cls}}(t)-\frac{z_{\text{el}}(t)}{{\left[z_{\text{el}}{\left(t\right)}^{2}+\alpha^{2}\right]}^{3/2}}.$$

The first term corresponds to the interaction with the cluster given by Equation 9. The second term stands for the force due to the interaction between photoemitted electron and the core hole left behind. The electron-hole interaction in our study is given by the regularized Coulomb potential *V*_el−*h*_:

(13) $$V_{\text{el}-h}=-\frac{1}{\sqrt{z_{\text{el}}{\left(t\right)}^{2}+\alpha^{2}}}.$$

We use *α*^2^ = 0.5 to avoid divergence at time *t *= 0.

## Results and discussion

In the following discussion, we center on the force experienced by the photoemitted electron due to the interaction with the cluster. This force is given by Equation 9 and allows us to study the important aspects of the electron density dynamics in the cluster. We study such force in the two approximations mentioned in the previous section for the motion of the (classical) photoemitted electron. In the first approximation, in which the electron moves with constant velocity, we can conveniently identify the effect of the hole screening on the movement of the photoemitted electron. To quantify the effect of the dynamic screening of the hole, we calculate the work performed by the force $F_{z}^{\text{cls}}$ along the electron trajectory. This quantity is directly linked with the energy loss of the ejected particle. In the second approximation, the velocity of the electron is allowed to vary according to Newton laws. This approximation might be closer to the real photoemission process. Also in this case, we find an important influence of the hole screening dynamics on the force experienced by the emitted electron and, thus, on the energy loss during the photoemission process.

### Constant velocity approximation

**Figure 2:**
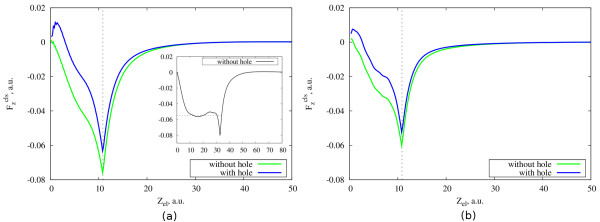
**Cluster-induced force acting on the photoemitted electron.** Cluster-induced force (Equation 9) acting on the electron moving away from the center of the cluster (*N *= 20, *r*_s_ = 4) as a function of the electron position. We consider the cases without and with a localized hole at the center of the cluster. The electron moves with a constant velocity: (**a**) *υ *= 1 a.u. (Inset, for cluster-induced force for a larger cluster with 556 electrons, the hole is not included. The horizontal dotted line corresponds to the stopping power in an homogeneous electron gas with *r*_s_ = 4.) and (**b**) *υ *= 1.5 a.u. Vertical dashed lines correspond to the radius of the cluster, *R*_cl_ = 10.86 a.u.

In the constant velocity approximation, we calculate the cluster-induced force in two different cases, namely, with a localized hole at the center of the cluster (potentials in Equations 6 and 7 are included in the calculations) and without the hole (only the potential in Equation 6 is included). In this approximation the direct interaction between the hole and electron is not included. In spite of this, we find that the presence of the hole modifies the electron dynamics in the cluster because there are two different charges to be screened. We have studied four Na clusters (*r*_s_ = 4) with 20, 58, 106, and 556 electrons. We consider three different velocities of the photoemitted electron: 1, 1.5, and 2.5 a.u. Figure 2 shows the cluster-induced force acting on the photoemitted electron as a function of the electron position in the cases of *υ *= 1 and *υ *= 1.5 a.u. for the small cluster with 20 electrons. From the present results, it follows that in case of the presence of the hole, the cluster-induced force on the photoemitted electron has a positive value at short times for the two chosen velocities. This indicates that the cluster response tends to accelerate the electron at the very beginning of its movement. The repulsive cluster-induced force is related to the screening of the hole. More precisely, at the beginning of its movement, when close to the hole, the electron is repelled from the hole vicinity by the cluster electrons that arrive to screen the hole. When the hole is not included in the calculation, the above effect is not observed, and the emitted electron is mainly decelerated all along its trajectory.^a^ This deceleration is due to two effects. First, within the cluster, the electron suffers the stopping characteristic of any charged particle moving in an electron gas [11]. Second, as the electron approaches the cluster surface, we can clearly see the contribution to the forces associated with the overcoming of the surface-potential step.

These two decelerating contributions are difficult to disentangle for very small clusters, like those in the main panels of Figure 2. However, the force experienced by a particle moving inside a large jellium cluster reaches a stationary regime and oscillates around a mean value. This can be seen in the inset of Figure 2a for a cluster containing 556 electrons. The mean value of the force is the so-called stopping power and only depends on the electron density. In the case of sodium (*r*_s_ = 4), the stopping power is around 0.055 a.u. for a negatively charged particle moving with a velocity of 1 a.u. [10].

**Figure 3:**
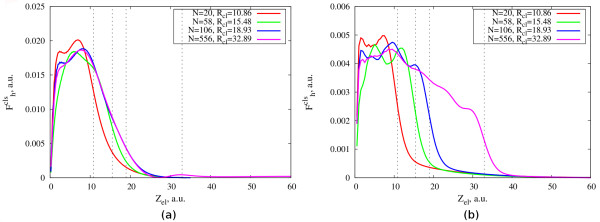
**Component of the cluster-induced force due to the hole screening.** Component of the cluster-induced force acting on the moving electron due to the dynamic screening of the hole by the electronic charge of the cluster, $F_{h}^{\text{cls}}(z)$. Results are shown as a function of the electron position for jellium clusters (*r*_*s *_= 4) of different size comprising *N *= 20, 58, 106, and 556 electrons. Electron velocities are (**a**) *υ *= 1 a.u, (**b**) *υ *= 2.5 a.u. Vertical dashed lines correspond to the radii of the clusters.

The influence of the hole screening on the moving electron can be better analyzed if we look at the difference of forces shown in the Figure 2: $F_{h}^{\text{cls}}(z)=F_{h,\text{el}}^{\text{cls}}(z)-F_{\text{el}}^{\text{cls}}(z)$. Here, $F_{h,\text{el}}^{\text{cls}}(z)$ ( $F_{\text{el}}^{\text{cls}}$) is the cluster-induced force on the photoemitted electron calculated with (without) explicit inclusion of the positive point charge at the center of the cluster. With this definition, $F_{h}^{\text{cls}}(z)$ is the force felt by the photoemitted electron specifically due to the cloud of electronic density that dynamically screens the hole. This quantity is shown in Figure 3 for all clusters considered and for two different velocities.

As it is seen from the graphs, the effect of the screening of the hole is larger in the case of the smaller electron velocity 1 a.u. in Figure 3a. This is related to the time the photoelectron spends in the neighborhood of the hole and to the characteristic time of the hole screening. The slow photoelectron stays near the hole long enough for the screening of the hole to be performed. Therefore, it experiences a large force due to the piling up of electronic charge around the hole. The fast electron, however, leaves the hole at short times which are not enough for a significant piling up of screening charge. Hence, the effect associated with the hole screening becomes smaller for higher electron velocities. It is worth noting that, for the slow electron, $F_{h}^{\text{cls}}(z)$ is almost identical for the two largest clusters considered here and it is very small for *z*_el_ > 25 a.u. Both observations indicate that the screening of the hole is well established and basically reaches its stationary value at the corresponding time scale. For the faster electron, however, the value of $F_{h}^{\text{cls}}(z)$ at large *z*_el_ is different for different cluster radii. This is linked to the time evolution of the screening density which still goes on by the time the electron reaches the cluster boundary. These conclusions are corroborated by the induced electron density dynamics plots discussed below.

In order to quantify the effect of the hole screening, we calculate the electron energy loss due to the interaction with the cluster electrons for the case of *N*=20 and for the velocities 1, 1.5, and 2.5 a.u. of the photoemitted electron. The energy loss *E*_loss_, given by Equation 8, is defined as the work performed by the cluster-induced force acting on the moving electron. The results are summarized in Table 1 in which the difference of the cluster-induced energy loss *ΔE*_loss_, with and without the hole, is also shown.

**Table 1:** **Estimated energy loss (Equation** 8**) for a photoemitted electron as a function of its velocity**

	***N *= 20**	***υ = *1 a.u.**	***υ = *1.5 a.u.**	***υ = *2.5 a.u.**
*E*_loss_, a.u.	Without hole	0.60	0.44	0.22
	With hole	0.37	0.30	0.16
*ΔE*_loss_ , a.u.		0.23	0.14	0.06

The electron is assumed to follow a trajectory with constant velocity. The difference in energy loss ***ΔE***_loss_ for the case of absence and presence of a hole in the center of the cluster is also given.

The presence of the hole reduces the cluster-induced energy loss for all velocities. The value of *ΔE*_loss_ also shows that the effect of the hole screening is more significant the slower the electron. The energy loss of the electron moving at 1 a.u. decreases almost by a factor of 2 when we include the hole screening in the process. An interesting consequence is that at low velocities, the effects associated with the hole screening might become crucial in determining if the photoemission process can indeed take place or not. For example, the kinetic energy of the slowest electron considered in Table 1 is 0.5 a.u. Since the energy loss in the case without hole is 0.6 a.u., this electron cannot be photoemitted from the cluster. However, in the presence of the hole, photoemission becomes possible.

The study of the electron dynamics during photoemission in the constant velocity approximation leads us to two conclusions: 1) the screening of the hole by the cluster electrons leads to a repulsive (accelerating) force acting on the photoemitted electron at the beginning of its movement; 2) the effect of the hole screening is reduced for faster (more energetic) photoemitted electrons.

### Varying velocity approximation

The results discussed so far are obtained using a simple model in which the photoemitted electron moves with a constant velocity. In a real photoemission process, however, the velocity varies due to the different elastic and inelastic forces acting on the electron. In order to be sure that none of the effects discussed above is an artifact of the model and to prove our conclusions, we simulate the photoemission process in a more realistic second approximation. In this approximation, the velocity and coordinate of the electron are dependent on time, according to Equations 10, 11, and 12. The electron and hole interact via a regularized Coulomb potential in Equation 13.

**Figure 4:**
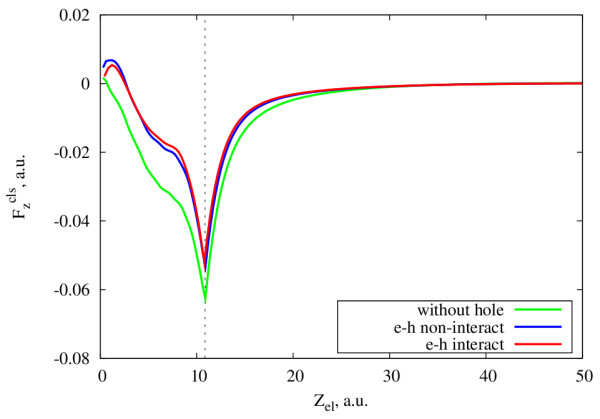
**Cluster-induced force acting on the electron moving away from the center of the cluster.** Cluster-induced force (Equation 9) acting on the electron moving away from the center of the cluster (*N *= 20, *r*_*s *_= 4), as a function of the electronic position in three cases: (1) There is no hole at the center of the cluster and the initial velocity of the electron is 1.5 a.u. (green line). (2) There is a hole at the center of the cluster, but the photoemitted electron does not interact directly with the hole. The initial velocity of the electron is 1.5 a.u. (blue line). (3) There is a hole at the center of the cluster and a direct electron-hole interaction (Equation 13) is included. The initial velocity of the electron is 2.25 a.u. (red line). The vertical dashed line shows the radius of the cluster, *R*_cl_ = 10.86 a.u.

In Figure 4 we show the results for the small cluster with 20 electrons in varying velocity approximation. The force felt by the moving electron due to the interaction with the cluster electrons (Equation 9) is calculated for three different cases. In the first case, we do not include the hole in the cluster. In the second case, there is a hole to be screened, but we do not include the direct interaction between the hole and the photoelectron when performing the trajectory calculation. In the third case, the direct interaction between the photoelectron and the hole is included. In the first two situations, the initial velocity of the electron is set to 1.5 a.u., while in the third situation it is set to 2.25 a.u. This difference in the velocity corresponds to the energy of the electron-hole interaction. Indeed, with the parameter *α*^2^ = 0.5 a.u. used in Equation 13, the ‘binding energy’^b^ of the electron is around 1.4 a.u. Taking into account this binding energy, the initial velocity of 2.25 a.u. for an electron photoemitted in vacuum and interacting with the hole leads to a final velocity of ∼ 1.5 a.u. Therefore, our choice of initial velocities allows a direct comparison of the two cases, with and without direct electron-hole interaction, in the case of photoemission inside the cluster.

One can see from Figure 4 that the behavior of the cluster-induced force in this more realistic model is similar to the simple model considered before (Figure 2). Whenever the hole screening is taken into account, there is an acceleration force acting on the electron at the beginning of its trip. Similar to the constant velocity approximation, when performing calculations along a more realistic trajectory with different launch velocities, we also found that the effect of the hole screening decreases when the initial velocity of the electron increases. Moreover, we found that the cluster-induced force is quite independent on whether electron and hole directly interact with each other or not. However, this is valid only if the final velocity of the photoemitted electron which interacts with the hole is equal to that which does not. This shows that as far as the final energy of the photoemitted electron is the same, the cluster-induced force acting on the photoemitted electron is mainly affected by the presence or absence of the hole screening, and not by the details of the electron trajectory nearby the hole.

### Time evolution of electronic density

**Figure 5:**
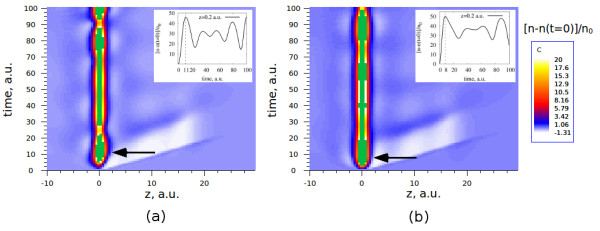
**Time evolution of the electronic density of the spherical cluster.** The induced electronic density is shown close to the symmetry *z*-axis (*ρ *= 0.02 a.u.,*z*). The time evolves along the vertical axis. The color map shows the change in density in units of the background density *n*_0_. The color scale is limited to a maximum value of 20 in order to reveal the effects in the regions where the induced density is small. The induced density above this value is shown in green. The actual maximum value of the induced density is around 50 in units of background density. It corresponds to the small *z* region around the position of the hole. (**a**) shows the results of the TDDFT calculation of the complete system. In (**b**) the induced density is calculated as a sum of two contributions (see text for the explanation). Cluster parameters are *r*_*s *_= 4, *N *= 106, *R*_cl_ = 18.93 a.u. The velocity of the electron is constant and is equal to *υ *= 1 a.u. Insets: profile of the plot along the time axis at (*ρ *= 0.02 a.u., *z* = 0.2 a.u.).

To continue the discussion on electron density dynamics in the cluster, we illustrate the effect of the coupling of both processes - dynamic screening of the hole and dynamic screening of the moving electron. Figure 5 shows the time evolution of the electronic density of the spherical cluster with *N *= 106 electrons. The hole and the electron are created at time *t *= 0 at the center of the cluster (*z* = 0), and the electron is moving along the positive part of the *z*-axis with a constant velocity of *υ *= 1 a.u. The induced electronic density close to the *z*-axis *Δn *=* n*(*ρ*_0_,*z*,*t*)−*n*(*ρ*_0_,*z*,0), where *ρ*_0_ = 0.02 a.u., is plotted in units of background density *n*_0_.

Figure 5a shows the results of a direct TDDFT calculation of the induced electronic density for the cluster with a static hole at the center and a photoelectron moving away from the center of the cluster along the *z*-axis. Figure 5b shows the induced electronic density obtained as a sum of two different contributions:

(14) $$\Delta n(t)=\delta n_{h}(t)+\delta n_{\text{el}}(t).$$

Here, *δn*_*h*_(*t*) is the TDDFT result for the induced electron density due to the appearance of only the localized hole at the center of the cluster. Similarly, *δn*_el_(*t*) is the TDDFT result for the induced electron density in response to a photoelectron moving from the center of the cluster where no hole is present. Therefore, Figure 5b shows a linear superposition of the electronic charges screening the static hole and moving photoelectron. In the inset of both graphs, we show the time evolution of the electronic density at a given point (*ρ *= 0.02 a.u., *z *= 0.2 a.u.).

The white area in the main plots shows a depletion of the electronic density in the cluster that roughly follows the trajectory of the electron. It is due to the Coulomb repulsion between the moving electron and the rest of the electrons in the cluster. The black arrows indicate the time at which the screening of the hole is fully developed, i.e., the induced electron density in the close vicinity of the hole roughly integrates to one. This time is also shown in the inset of each plot and is equal to 11 and 8 a.u. for the cases in Figure 5a,b. Thus, there is a delay in the TDDFT screening of the hole as compared to the linear superposition case. Moreover, comparing the charge distribution for negative and positive values of *z*, we can see a clear asymmetry in the screening charge for the TDDFT calculation with both hole and electron simultaneously included. This asymmetry is absent in Figure 5b, corresponding to the linear superposition of electron and hole separate screenings, and clearly indicates that the dynamics of the hole screening is affected by the presence and movement of the emitted electron. Therefore, we can conclude that the TDDFT calculation, considering both the hole at the center and the electron photoemitted from the center of the cluster, includes a combined effect of the dynamic screening of both particles in the relaxation processes in the cluster. This combined effect is also visible in the oscillations of the electronic density, where the periods of these oscillations are slightly different for the two cases considered.

## Conclusions

In this study a semi-classical model was used to describe the dynamic screening of a moving photoelectron and a localized core hole left behind as a result of the interaction of XUV pulses with small metal clusters. The motion of the photoemitted electron is described classically, and the electron dynamics in the clusters is studied using the time-dependent density functional theory.

We have shown that when the hole is explicitly included in the calculation, the photoemitted electron is accelerated by the cluster electrons that pile up nearby the cluster center to dynamically screen the hole. This effect is observed by comparing the forces acting on the photoemitted electron due to the interaction with the cluster in which a hole is present or absent at the center. In order to quantify the effect of the hole screening, we have calculated the energy loss of the photoelectron. We have shown that the presence of the hole reduces significantly the cluster-induced energy loss and that this effect is velocity-dependent. The higher is the energy of the photoemitted electron, the smaller is the effect induced by the hole screening.

These conclusions were obtained using a relatively simple approximation in which the photoemitted electron moves with constant velocity. The conclusions are proven to remain valid when the interaction between photoemitted electron and core hole left behind is included in the calculation, and the velocity of the electron is allowed to vary with time. We have illustrated the time evolution of the electron density in the cluster during the photoemission process, and we have shown that the TDDFT calculation allows us to see the coupled effect of the screening of both the hole and the electron in the relaxation processes inside the cluster.

The semi-classical model used here allows for a detailed analysis of the effect of the dynamic screening of the hole. However, the simplicity of the model and the classical treatment of the photoemitted electron also prevent a direct translation of our findings to the experimental situation. Thus, a clear understanding of the implications of the present results for photoemission experiments is still an open question that requires further work.

## Endnotes

^a^ In Figure 2b one can see that even in the calculation without hole, the force acting on the photoemitted electron exhibits very small positive values at the beginning of the electron trajectory. In this case, this is not related to the dynamic processes inside the cluster, but to the slightly inhomogeneous distribution of the electron density in the small clusters. The ground state electronic density of a cluster with 20 electrons and *r*_*s*_=4 is shown in Figure 1a. The electron density shows a maximum at the center, where it is larger than the value of the positive background density. Since we start the dynamical calculations using the ground state density, this leads to a positive (i.e., repulsive) value of the initial force acting on a negatively charged particle located nearby the center of the cluster.^b^ Binding energy here means the interaction energy when both the electron and the hole are located at the center of the cluster.

## Abbreviations

JM: Jellium model; KS: Kohn-Sham; TDDFT: Time-dependent density functional theory; XUV: Extreme ultraviolet.

## Competing interests

The authors declare that they have no competing interests.

## Author’s contributions

All authors have made substantial contributions to the conception, acquisition and interpretation of data. All authors have been involved in drafting the manuscript. DSP, AGB, and RDM have been revising the manuscript for important intellectual content and have given final approval of the version to be published. All authors read and approved the final manuscript.

## References

[B1] Schattke W Van Hove MA(Eds) Solid-State Photoemission and Related Methods: Theory and Experiment 2003 Berlin: Wiley-VCH

[B2] Cavalieri AL Müller N Uphues Th Yakovlev VS Baltuska A Horvath B Schmidt B Blümel L Holzwarth R Hendel S Drescher M Kleineberg U Echenique PM Kienberger R Krausz F Heinzmann U Attosecond spectroscopy in condensed matter Nature 2007 449 1029 1032 10.1038/nature06229 17960239

[B3] Díez Muiño R Sánchez-Portal D Silkin VM Chulkov EV Echenique PM Time-dependent electron phenomena at surfaces P Natl Acad Sci USA 2011 108 971 10.1073/pnas.1008517107 PMC3024703 21098300

[B4] Kazansky AK Echenique PM One-electron model for the electronic response of metal surfaces to subfemtosecond photoexcitation Phys Rev Lett 2009 102 177401 19518828 10.1103/PhysRevLett.102.177401

[B5] Lemell C Solleder B Tőkési K Burgdörfer J Simulation of attosecond streaking of electrons emitted from a tungsten surface Phys Rev A 2009 79 062901

[B6] Krasovskii EE Attosecond spectroscopy of solids: streaking phase shift due to lattice scattering Phys Rev B 2011 84 195106

[B7] Alonso JA Structure And Properties of Atomic Nanoclusters 2006 London: Imperial College Press

[B8] Borisov AG Sánchez-Portal D Díez Muiño R Echenique PM Building up the screening below the femtosecond scale Chem Phys Lett 2004 387 95 100 10.1016/j.cplett.2004.01.114

[B9] Borisov AG Sánchez-Portal D Díez Muiño R Echenique PM Dimensionality effects in time-dependent screening Chem Phys Lett 2004 393 132 137 10.1016/j.cplett.2004.06.026

[B10] Quijada M Borisov AG Díez Muiño R Time-dependent density functional calculation of the energy loss of antiprotons colliding with metallic nanoshells Phys Stat Sol 2008 205 1312 1316 10.1002/pssa.200778157

[B11] Quijada M Borisov AG Nagy I Díez Muiño R Echenique PM Time-dependent density-functional calculation of the stopping power for protons and antiprotons in metals Phys Rev A 2007 75 042902

[B12] Gadzuk JW S̆unjić M Excitation energy dependence of core-level x-ray-photoemission-spectra line shapes in metals Phys Rev B 1975 12 524 10.1103/PhysRevB.12.524

[B13] Noguera C Spanjaard D Friedel J Dynamic screening of a core hole: I. Semiclassical model J Phys F: Metal Phys 1979 9 1189 10.1088/0305-4608/9/6/022

[B14] Jänkälä K Tchaplyguine M Björneholm O Huttula M Mikkelä M-H Photon energy dependent valence band response of metallic nanoparticles Phys Rev Lett 2011 107 183401 22107629 10.1103/PhysRevLett.107.183401

[B15] Ashcroft NW David Mermin N Chapter 1 The Drude theory of metals In Solid State Physics 1976 Philadelphia: Saunders College Publishing 5 5

[B16] Hohenberg P Kohn W Inhomogeneous electron gas Phys Rev 1964 136 B864 B871 10.1103/PhysRev.136.B864

[B17] Kohn W Sham LJ Self-consistent equations including exchange and correlation effects Phys Rev 1965 140 A1133 A1138 10.1103/PhysRev.140.A1133

[B18] Perdew JP Zunger A Self-interaction correction to density-functional approximations for many-electron systems Phys Rev B 1981 23 5048 10.1103/PhysRevB.23.5048

[B19] Ekardt W Work function of small metal particles: self-consistent spherical jellium-background model Phys Rev B 1984 29 1558 10.1103/PhysRevB.29.1558

[B20] De Heer WA The physics of simple metal clusters: experimental aspects and simple models Rev Mod Phys 1993 65 611 10.1103/RevModPhys.65.611

[B21] Knight WD Clemenger K de Heer W A Saunders WA Chou MYandCohen ML Electronic shell structure and abundances of sodium clusters Phys Rev Lett 2141 52 1984

[B22] Runge E Gross EKU Density-functional theory for time-dependent systems Phys Rev Lett 1984 52 997 10.1103/PhysRevLett.52.997

[B23] Borisov AG Juaristi JI Díez Muiño R Sánchez-Portal D Echenique PM Quantum-size effects in the energy loss of charged particles interacting with a confined two-dimensional electron gas Phys Rev A 2006 73 012901

[B24] Borisov AG Gauyacq JP Shabanov SV Wave packet propagation study of the charge transfer interaction in the F−-Cu(111) and -Ag(111) systems Surf Sci 2001 487 243 257 10.1016/S0039-6028(01)01102-5

[B25] Chulkov EV Borisov AG Gauyacq JP Sánchez-Portal D Silkin VM Zhukov VP Echenique PM Electronic excitations in metals and at metal surfaces Chem Rev 2006 106 4160 4206 10.1021/cr050166o 17031983

